# Aloof Electron Probing of In-Plane Surface Photovoltaic Charge Distributions on GaAs Surfaces

**DOI:** 10.1021/acsphotonics.4c01997

**Published:** 2025-01-27

**Authors:** Zilin Chen, Wayne Cheng-Wei Huang, Herman Batelaan

**Affiliations:** ‡ Department of Physics and Astronomy, Northwestern University, Evanston, Illinois 60208, United States; § Department of Physics and Astronomy, University of Nebraska-Lincoln, Lincoln, Nebraska 68588, United States; ∥ Department of Physics, National Tsing Hua University, Hsinchu 30013, Taiwan (R.O.C.)

**Keywords:** aloof electron deflection, diffracted electron wave, surface photovoltaic charge, oxide-induced surface trapping state, undoped GaAs, impeded photocarrier recombination

## Abstract

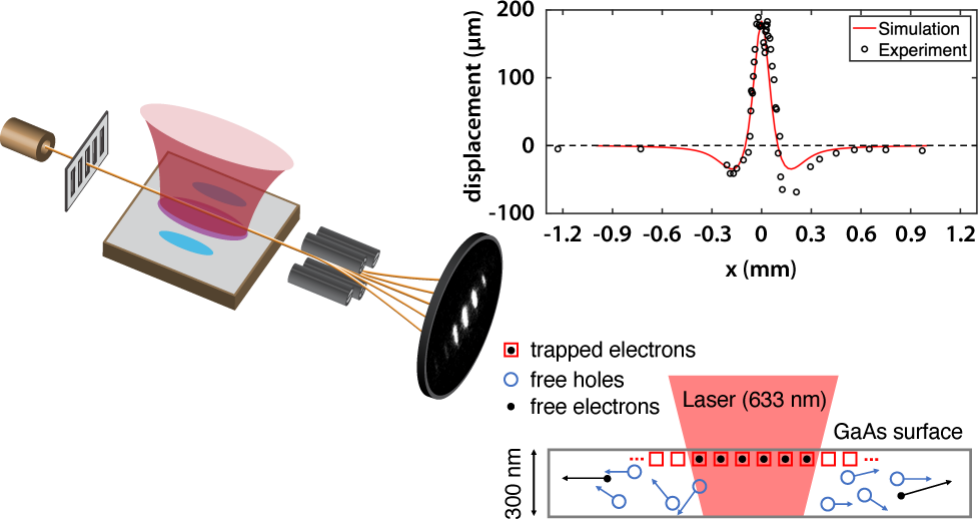

The motion of free electrons moving parallel and above a semiconductor surface can be influenced by shining a laser light onto the surface. Here we report strong deflection of aloof electrons by an undoped GaAs surface illuminated with a 633 nm laser. The deflecting electric field from the surface photovoltaic charges extends 100 μm into the vacuum. As surface photovoltage (SPV) is sensitive to the electronic states of the GaAs surface, the aloof electron beam serves as a probe for SPV charge dynamics on the mesoscopic length scale. The observed in-plane SPV charge distribution persists beyond 1 second after the laser beam is blocked. Our work suggests the possibility of writing designed 2D charge patterns on semiconductor surfaces with a scanning laser beam, providing unusual flexibility for electron beam manipulation.

## I Introduction

The surface photovoltage (SPV) can facilitate electrostatic near fields above optically illuminated semiconductor surfaces. The near fields are important for SPV spectroscopy,^1^ for understanding charge separation and recombination processes in solar cells,^2^ for electron beam manipulation,^3^ and for studying quantum decoherence and dissipation of free electrons.^4,5^ In this study, we probe the SPV near fields on an undoped single-crystalline GaAs (110) surface through the deflection of aloof electrons. We observe strong deflection when the surface is under superband or subband photoexcitation. As SPV near fields depend on the transport properties and electronic states of the surface, our approach serves as a probe for the SPV charge dynamics.

Recent developments on SPV measurement include SPV microscopy^2^ and scanning ultrafast electron microscopy.^6^ These methods are direct probes of the 2D photovoltaic charge distribution on the microscopic scale. Our approach complements the existing methods in that we measure the 3D SPV near fields on the mesoscopic length scale. Specifically, the vertical electron deflection due to SPV near fields is proportional to the 1D integral of the SPV charge distribution along the direction of electron beam propagation.^7^ The first use of electron deflection to image surface charge distribution was done in the context of laser ablation,^8^ with a maximum deflection magnitude around 100 μm. The vertical deflection we report here is up to 200 μm (7 beam diameters) at 40 cm after the GaAs surface. It can thus be used with an aperture to switch an electron beam on and off. Also, the SPV near field extends 100 μm into the vacuum, providing a long working distance. Our method is robust, requires no nanofabrication, and works at modest vacuum (10^–6^ Torr).

The manipulation of free electrons by laser-illuminated material structures is of general interest, and many examples exist for such technology. For example, the electron beam, and many copies thereof, can be steered by laser light in the presence of a surface and used for multibeam electron lithography.^3^ In addition, control of electron motional states in dielectric laser accelerators,^9^ control of attosecond electron dynamics near a nanotip,^10,11^ and laser-induced phase modulation of an electron wave^12^ are but a few examples of exquisite motion control through photoinduced near fields. As SPV charge distributions are closely linked to the intensity profile of the laser light, simulation can be used to guide the development of a designed near field structure, providing another useful tool for optical control of free electrons.

A detailed understanding of photoinduced near fields and the ensuing change of surface resistivity are also needed for attaining controlled electron-surface decoherence.^13−16^ This approach was first proposed by Zurek,^4^ intended for testing Caldeira and Leggett’s quantum dissipation theory.^5^ In our experiment, we found that the electron diffraction pattern can be strongly distorted by the gradient force of the SPV near field, but the beam coherence is not affected by the light-modulated surface resistivity.

## II Results and Discussions

### A Superband Photoexcitation

**Figure 1:**
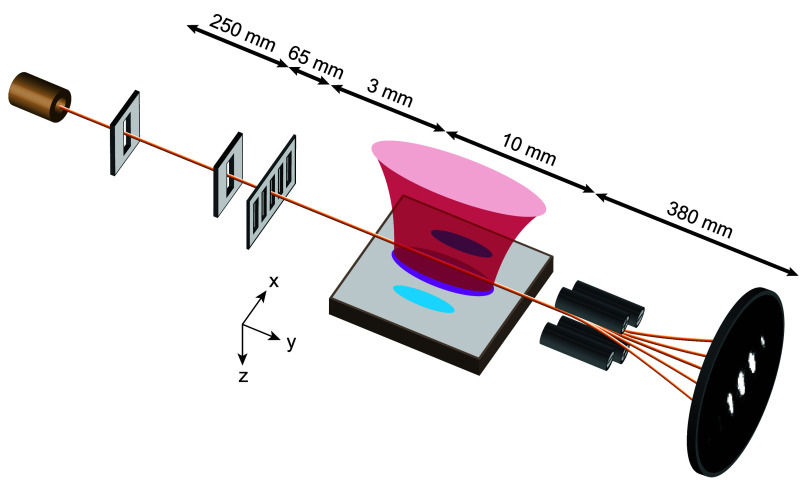
Schematic of the electron deflection experiment. A thermionic electron gun (top left) emits electrons at an energy of 1.67 keV. Two collimation slits separated by 250 mm limit the transverse momentum spread of the beam and deliver an electron beam (yellow) with a transverse spatial coherence of ∼500 nm to a nanofabricated grating. The grating has a periodicity of 100 nm and transmits 50% of the electron beam. The diffracted electron beam passes parallel to and over a 10 mm long undoped GaAs surface (gray). The surface is illuminated with an elliptically shaped laser beam (red). The laser beam widths parallel and transverse to the direction of electron beam propagation are 5 mm and 100 μm, respectively. The transverse laser beam width is significantly larger than the spatial extent of the diffracted electron beam. The laser wavelength is 633 nm for superband excitation or 1064 nm for subband excitation. The nominal laser power is 1 mW. The laser produces a SPV charge distribution (blue ovals) whose electrostatic near field deflects the free electrons at an electron-surface distance up to 100 μm. The deflected electron beam is magnified with a quadrupole lens and recorded with an imaging detector (bottom right).

In our experiment (Figure 1), in-plane SPV charge distributions are probed with a diffracted electron beam through vertical beam displacement. We use rate equations to model the photovoltaic carrier dynamics^1,17^ and an electron trajectory simulation^16^ to compute the near-field interaction that leads to the beam displacement.

**Figure 2:**
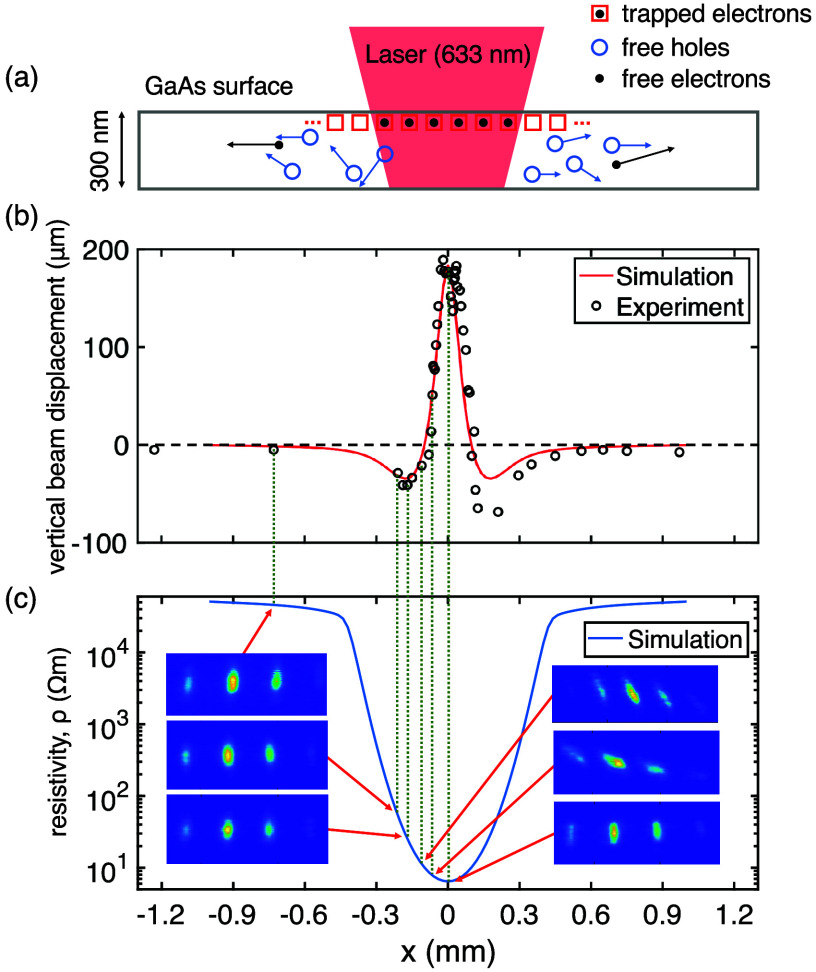
Superband photoexcitation at the GaAs surface. (a) A schematic of superband excitation. (b) The vertical displacement of a diffracted electron beam is measured (black circles) relative to the light-off position (black dashed line) as the laser beam is moved along the *x*-axis. Here, the electron-surface distance is 13 μm. In the central region, the deflection is away from the surface due to the repulsive force from the trapped electrons. The superband model together with an electron trajectory simulation (red solid line) gives good agreement with the experiment. (c) The superband model predicts a spatially modulated resistivity (blue solid line) with a four-orders-of-magnitude variation across the laser-illuminated area. At the positive-negative crossover point of the SPV charge distribution, the field direction changes from vertical to horizontal, and the electron diffraction patterns (colored insets) exhibit varying degrees of rotation without changing the contrast. The charge distribution (not shown) is situated at the top of the inset.

As the undoped GaAs has a bandgap of 1.42 eV (873 nm), illuminating the surface with a 633 nm continuous-wave He-Ne laser creates free electrons and holes through superband excitation. The generated electrons and holes are close to the surface because the laser penetration depth in this case is only 300 nm.^18^ This results in most free electrons being trapped in the oxide-induced surface states,^19,20^ while the free holes diffuse to the surroundings (Figure 2a). Prior to photocarrier injection, the surface states are mostly unoccupied because of the low intrinsic carrier density (∼10^6^ cm^–3^) in undoped GaAs.^21^ In the equilibrium state, with the laser continuously on, the internal electric field within the charge distribution becomes strong enough to keep the holes from diffusing further away. The ensuing charge distribution has trapped electrons in the laser-illuminated area and free-holes at the surroundings, as also indicated by the electron deflection data (Figure 2b). The corresponding band bending is spatially modulated in the in-plane direction, in contrast to the typical scenario, where the band bending is homogeneous across the surface. Variation in the local surface atomic structures could suppress or enhance local band bending^22^ and result in the asymmetry present in Figure 2b. The magnitude of electron deflection saturates at a low laser power (1 mW) and is independent of laser polarization.

We use rate equations^1,17,23^ to obtain the equilibrium density distributions for free electron *n*(*x*), free hole *p*(*x*), and trapped electron *n*_*t*_(*x*),
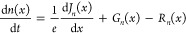
1
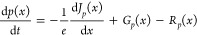
2
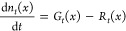
3where *G*_*i*_(*x*) and *R*_*i*_(*x*) denote the generation and recombination rates, respectively, and *e* is the electron charge unit. The charge current density *J*_*i*_(*x*) depends on the balance between diffusion and the drift caused by the internal electric field *E*(*x*),
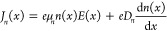
4
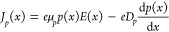
5where  and  are the mobilities of free electrons and holes in undoped GaAs.^21^ The corresponding diffusion coefficients are  and  according to the Einstein relation. The internal electric field *E*(*x*) is determiend by the total charge density ρ(*x*) = *e*(*p*(*x*) – *n*(*x*) – *n*_*t*_(*x*)). The thermal recombination rate  in eq 1 is the sum of the thermal transition from the conduction band to the valence band  and the thermal capture rate from the conduction band to the surface trapping states , where *N*_*t*_ is the trapping state density.^1^ On the other hand, the generation rate  has four contributions. The  and  terms correspond to thermal and optical excitation from the valence band to the conduction band. Similarly, the  and  terms characterize thermal and optical excitation rates from the trapping states to the conduction band. These four terms are given by
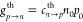
6a
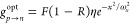
6b
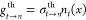
6c
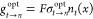
6dwhere *n*_0_ and *p*_0_ are the intrinsic carrier densities (∼10^6^ cm^–3^) in the absence of laser illumination, *F* is the laser photon flux (∼10^18^ cm^–2^ s^–1^), *R* = 0.3 is the GaAs reflectivity at 633 nm,^24^ η = 0.7 is the quantum efficiency,^25^ ω_*x*_ = 100 μm is the laser beam width transverse to the direction of electron beam propagation,  and  are thermal and optical excitation rates from the trapping states to the conduction band. In eqs 2 and 3, the corresponding generation and recombination rates are
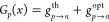
7a
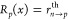
7b
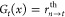
7c
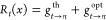
7d

We obtain the total charge density ρ(*x*) by solving the coupled rate eqs (eqs 1–3) with the parameters given above. To compare the SPV charge distribution ρ(*x*) with the electron deflection data, we construct an electron trajectory simulation^16^ using an electrostatic field derived from the charge distribution. In Figure 2b, we find good agreement between our model and the deflection data.

The superband model also predicts slow relaxation of the SPV charge distribution after the laser beam is blocked. While the SPV charge distribution is usually established in microseconds or faster,^6,7^ its relaxation can take seconds due to surface trapping states.^17,26^ After blocking the laser with a mechanical chopper, we observe that the electron deflection persists for 1 second. This opens an interesting prospect in which scanning a laser beam at a rate above 10 Hz can result in a “programmable” 2D surface charge pattern. The resulting SPV near field and its gradient may be exploited for building deformable electron-optical elements, such as electrostatic lenses or deflectors.

### B Subband Photoexcitation

**Figure 3:**
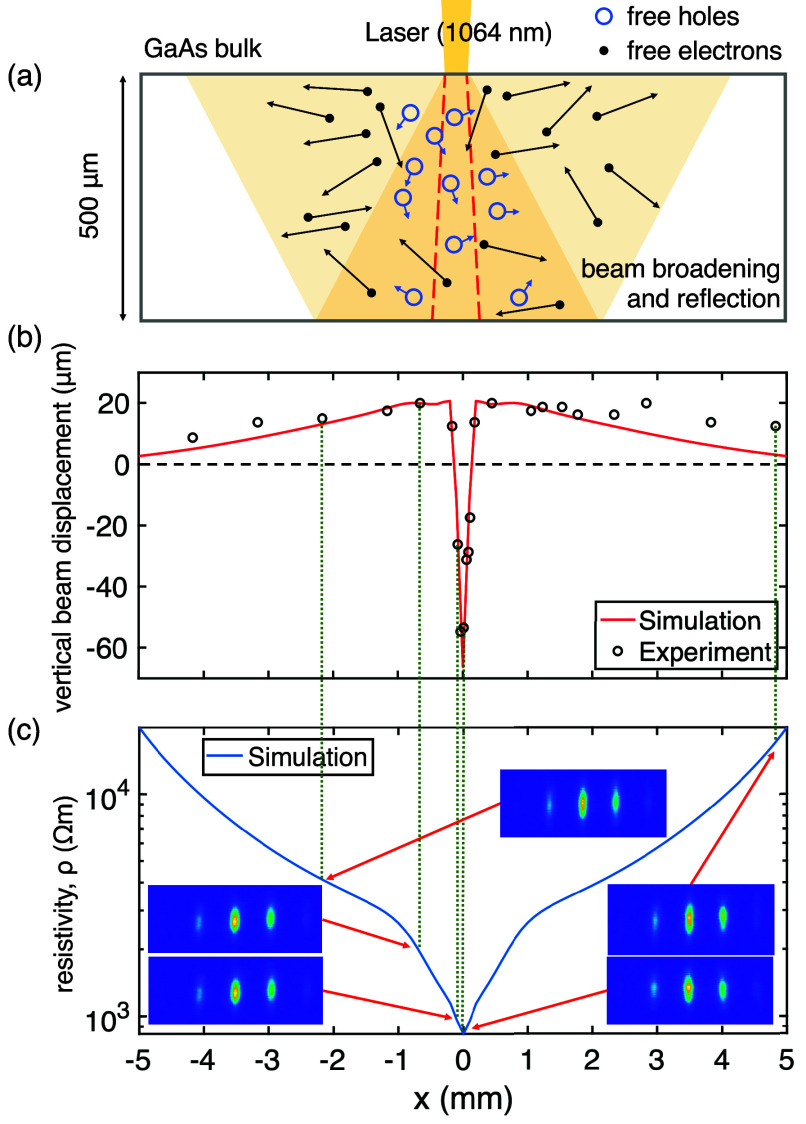
Subband photoexcitation in the GaAs bulk. (a) Schematic of subband excitation. The 1064 nm laser can penetrate deep into the material and create free electrons and holes in the bulk. Beam broadening and reflection from the back side (yellow shades) extend the carrier generation region to millimeters. The waist of the unobstructed laser beam is symbolically indicated with red dashed lines. (b) The vertical displacement of a diffracted electron beam is measured (black circles) relative to the light-off position (black dashed line) as the laser beam is moved along the *x*-axis. Comparison between the experiment and the subband model (red solid line) shows good agreement. In the central region, the deflection is toward the surface due to the attractive force from the free holes. Note that free electrons diffuse throughout the full length of the GaAs sample (10 mm). The electron-surface distance is 13 μm. (c) The laser-modulated surface resistivity (blue solid line) is given by the subband model. The electron diffraction patterns only exhibit mild distortion because the near-field gradient is weak.

In order to verify the connection between slow relaxation and surface trapping states, we illuminated the GaAs surface with a 1064 nm continuous-wave laser. The 1064 nm laser can penetrate deep into the material, because direct absorption by valence electrons is energetically unfavorable. Meanwhile, generation of free electrons and holes can still be achieved via EL2 defect-assisted subband photoexcitation.^27,28^ This allows for measurements of the SPV relaxation time in the absence of surface trapping states. We estimate the penetration depth of the 1064 nm laser to be >500 μm, as we observe a broadened laser spot at the back side of our GaAs sample (Figure 3a).

Generation of photocarriers in the bulk, where unoccupied surface trapping states are absent, can lead to, as a result of the Dember effect,^29^ a negative charge distribution throughout the bulk and a positive space charge distribution below the laser-illuminated area. This is shown in the electron deflection data as long side wings of negative charges and a relatively narrow center peak of positive charges (Figure 3b). The nonzero integral^7^ of the deflection curve implies a negative net charge at the surface. Given that charge carriers are generated throughout the bulk and a nonzero-sum charge distribution resides at the surface, the dimension perpendicular to the surface and the bulk charge carriers need to be included in the subband model. The free electron densities *n*_*s*,*b*_(*x*) associated with the surface and bulk bands are directly coupled through a vertical diffusion. The free hole densities *p*_*s*,*b*_(*x*) of the surface and bulk bands are also coupled in the same way. Meanwhile, the internal electric field *E*(*x*) responsible for the horizontal drift of free charge carriers in each band is determined by the total charge carrier density *p*_*s*_(*x*) + *p*_*b*_(*x*) – *n*_*s*_(*x*) – *n*_*b*_(*x*). Since surface trapping states are ignored in the subband model, here the generation and recombination rates do not depend on ,  and . Both primary and broadened laser beams are used in the simulation for photoexcitation of the surface and bulk bands. A comparison between our model and the electron deflection data shows good agreement (Figure 3b). As we modulate the laser intensity with a mechanical chopper, we determine the upper bound of the SPV relaxation time to be 0.6 ms.

## III Conclusion

In summary, we perform an electron deflection experiment with an undoped GaAs (110) surface. The surface is optically illuminated by low-power lasers for either superband or subband photoexcitation. We found good quantitative agreement between our experimental results and existing photovoltaic models. For 633 nm superband excitation, we observe a narrow central region populated with trapped electrons and equally narrow side wings of free holes. The electrostatic near field of SPV charges can cause vertical deflection of a diffracted electron beam at an electron-surface distance up to 100 μm. The presence of unoccupied surface trapping states significantly impedes photocarrier recombination at the surface, making the lifetime of the in-plane SPV charge distribution exceed over 1 second. For 1064 nm subband excitation, where no surface trapping states are involved, the SPV relaxation time is measured to be shorter than 0.6 ms. It is perhaps interesting to contemplate scanning a laser beam so that a designed 2D charge pattern could be “written” on the undoped GaAs surface via superband excitation. The gradient of the SPV near field can act as an electrostatic lens for the electron beam passing over the surface. Such “programmable” electron-optical elements may add an interesting approach to the optical control of free electrons.
